# Seroprevalence of SARS-CoV-2 Antibodies and Factors Associated with Seropositivity at the University of Salamanca: The DIANCUSAL Study

**DOI:** 10.3390/jcm10153214

**Published:** 2021-07-21

**Authors:** Antonio Muro, Moncef Belhassen-García, Juan Luís Muñoz Bellido, Helena Lorenzo Juanes, Belén Vicente, Josué Pendones, José Adserias, Gonzalo Sánchez Hernández, Miguel Rodríguez Rosa, José Luis Vicente Villardón, Javier Burguillo, Javier López Andaluz, Jose Angel Martín Oterino, Francisco Javier García Criado, Fausto Barbero, Ana Isabel Morales, Purificación Galindo Villardón, Rogelio González Sarmiento, on behalf of the DIANCUSAL Team

**Affiliations:** 1 Infectious and Tropical Diseases Group (e-INTRO), Institute of Biomedical Research of Salamanca-Research Center for Tropical Diseases at the University of Salamanca (IBSAL-CIETUS), Faculty of Pharmacy, University of Salamanca, 37008 Salamanca, Spain; belvi25@usal.es; 2 Institute of Biomedical Research of Salamanca (IBSAL), University of Salamanca, University Hospital of Salamanca, 37007 Salamanca, Spain; jlmubel@usal.es (J.L.M.B.); hlorenzojuanes@gmail.com (H.L.J.); jpendones@saludcastillayleon.es (J.P.); jamarot@usal.es (J.A.M.O.); fjgc@usal.es (F.J.G.C.); amorales@usal.es (A.I.M.); gonzalez@usal.es (R.G.S.); 3 Microbiology & Parasitology Service, University Hospital of Salamanca, 37007 Salamanca, Spain; 4 Department of Biomedical and Diagnostic Sciences, University of Salamanca, 37008 Salamanca, Spain; 5 IT Department, University of Salamanca Foundation (FGUSAL), University of Salamanca, 37008 Salamanca, Spain; huesca@usal.es; 6 Data Processing Center (CPD), University of Salamanca, 37008 Salamanca, Spain; gsh@usal.es; 7 Department of Statistics, University of Salamanca, 37008 Salamanca, Spain; miguel_rosa90@usal.es (M.R.R.); villardon@usal.es (J.L.V.V.); pgalindo@usal.es (P.G.V.); 8 Department of Chemistry-Physics, Faculty of Pharmacy, University of Salamanca, 37008 Salamanca, Spain; burgi@usal.es; 9 Department of Nursing and Phisiotherapy, University of Salamanca, 37008 Salamanca, Spain; j.andaluz10_11@usal.es (J.L.A.); fausbar@usal.es (F.B.); 10 Department of Internal Medicine, Faculty of Medicine, University Hospital of Salamanca, 37008 Salamanca, Spain; 11 Toxicology Unit, University of Salamanca, 37008 Salamanca, Spain; 12 Molecular Medicine Unit, Department of Medicine, University of Salamanca, 37008 Salamanca, Spain

**Keywords:** SARS-CoV-2, COVID-19, antibodies, seroprevalence, screening, university, Salamanca, Spain

## Abstract

Background: Systematic screening for antibodies against SARS-CoV-2 is a crucial tool for surveillance of the COVID-19 pandemic. The University of Salamanca (USAL) in Spain designed a project called “DIANCUSAL” (Diagnosis of New Coronavirus, COVID-19, in University of Salamanca) to measure antibodies against SARS-CoV-2 among its ~34,000 students and academic staff, as the influence of the university community in the spread of the SARS-CoV-2 pandemic in the city of Salamanca and neighboring towns hosting USAL campuses could be substantial. Objective: The aim of this study was to estimate the prevalence of SARS-CoV-2 antibodies among USAL students, professors and staff and to evaluate the demographic, academic, clinical and lifestyle and behavioral factors related to seropositivity. Methodology: The DIANCUSAL study is an ongoing university population-based cross-sectional study, with the work described herein conducted from July–October 2020. All USAL students, professors and staff were invited to complete an anonymized questionnaire. Seroprevalence of anti-SARS-CoV-2 antibodies was detected and quantified by using chemiluminescent assays for IgG and IgM. Principal findings: A total of 8197 (24.71%) participants were included. The mean age was 31.4 (14.5 SD) years, and 66.0% of the participants were female. The seroprevalence was 8.25% overall and was highest for students from the education campus (12.5%) and professors from the biomedical campus (12.6%), with significant differences among faculties (*p* = 0.006). Based on the questionnaire, loss of smell and fever were the symptoms most strongly associated with seropositivity, and 22.6% of seropositive participants were asymptomatic. Social distancing was the most effective hygiene measure (*p* = 0.0007). There were significant differences in seroprevalence between participants with and without household exposure to SARS-CoV-2 (*p* = 0.0000), but not between students who lived in private homes and those who lived in dormitories. IgG antibodies decreased over time in the participants with confirmed self-reported COVID-19 diagnoses. Conclusions: The analysis revealed an overall 8.25% seroprevalence at the end of October 2020, with a higher seroprevalence in students than in staff. Thus, there is no need for tailored measures for the USAL community as the official average seroprevalence in the area was similar (7.8% at 22 June and 12.4 at 15 November of 2020). Instead, USAL members should comply with public health measures.

## 1. Introduction

In December 2019, a novel coronavirus, severe acute respiratory syndrome coronavirus 2 (SARS-CoV-2), emerged that causes the illness designated COVID-19 and that has had devastating socioeconomic and public health consequences [1]. A pandemic was declared by the WHO in March 2020 after rapid human-to-human transmission and the intercontinental spread of the virus. More than 121 million people have been infected worldwide, with more than 3.0 million deaths [2]. In Spain, the first SARS-CoV-2 case was identified on 31 January and was determined to have been imported from Germany. Since then, the number of cases has increased rapidly in the country, and Spain is now one of the European countries most affected by the COVID-19 pandemic [3].

Control measures such as the use of masks, physical distancing, contact tracing and isolation in terms of people who have tested positive have been advised. However, these actions have been variably implemented and have proven insufficient in impeding the spread of COVID-19. Systematic screening for antibodies against SARS-CoV-2 is a crucial tool for surveillance of the pandemic and to predict when herd immunity might be reached [4]. A sero-epidemiological study provides information on the proportion of the population exposed and, if the antibodies are a marker of total or partial immunity, the amount of the population that remains susceptible to the virus. Since there is limited access to diagnostic tests, serological surveys are a valuable tool to assess the extent of the epidemic [5], and they have an advantage over epidemiological surveillance of confirmed COVID-19 cases that captures only a proportion of all infections. Seroprevalence studies have been conducted since the onset of the pandemic, mostly with health workers and the general population [6,7]. A nationwide, population-based sero-epidemiology study called “ENE-COVID” was carried out to analyze the prevalence of SARS-CoV-2 in Spain, showing remarkable differences between higher- and lower-prevalence areas [3].

Few seroprevalence studies have been carried out in academic institutions such as universities, and those that have been carried have been conducted with small cohorts [8,9,10,11,12]. Such studies are needed because university communities (faculty, staff and students) could be expected to be among the groups most exposed to SARS-CoV-2. In April 2020, the World Bank estimated that universities and other tertiary educational institutions were closed in 175 countries and communities and that studies were ended or significantly disrupted due to COVID-19 for more than 220 million post-secondary education students [13]. Spain was one of the countries with the strictest conditions during the first wave (March–April 2020), and leaving home was allowed only for essential needs. All universities were closed, and classes continued online with support from academic services.

The University of Salamanca (USAL) is located in western Spain and has ~30,000 students and over 3000 academic staff. USAL comprises a main campus in the city of Salamanca (40°50′0″ N, 6°0′0″ W) and three smaller campuses in Avila (40°39′15.65″ N, 4°41′46.4″ W), Zamora (41°45′0″ N, 6°0′0″ W) and Bejar (40°23′5″ N, 5°45′43″ W), all of which were assessed in this study. The ratio of students to Salamanca city inhabitants is 1:5, which is higher than that of other cities in Spain. Therefore, the influence of the university community in the spread of the SARS-CoV-2 pandemic in Salamanca could be substantial.

To measure antibodies in the university community, teams of professor and student volunteers from the health sciences faculties of USAL conducted the DIANCUSAL project. The aim was to characterize the university community to provide a basis for the eventual implementation of strategies to mitigate cases of COVID-19 at USAL. Thus, the primary objective was to estimate the prevalence of SARS-CoV-2 antibodies among members of USAL and compare it with that of the general population. The secondary objective was to evaluate the demographic, academic, clinical and lifestyle and behavioral factors related to seropositivity detection among members of the USAL community.

## 2. Material and Methods

### 2.1. Study Design and Population

**Figure 1:**
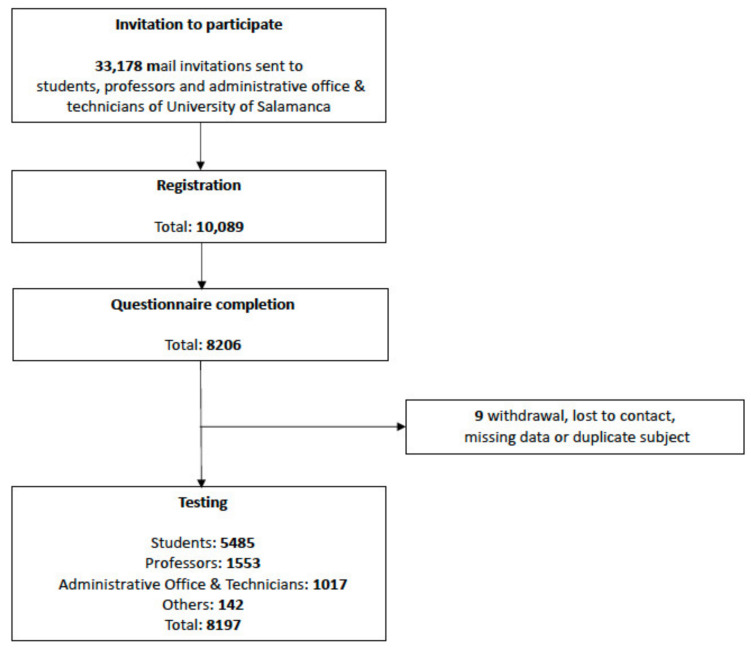
Participant flowchart through the recruitment process with eligibility screening, questionnaire completion and testing.

The DIANCUSAL (Diagnosis of New Coronavirus, COVID-19, in University of Salamanca) project is an ongoing university population-based cross-sectional study. A total of 33,178 students, professors, and staff at USAL were invited by e-mail to enroll in the study. The participants were volunteers and registered online. The flow chart in Figure 1 indicates the inclusion and exclusion criteria. The participants were enrolled in the current study on 14 July and participated until 30 October 2020.

### 2.2. Data Collection

The anonymized questionnaire focused on COVID-19 was available online on a website designed specifically for DIANCUSAL, https://diancusal.usal.es (accessed on 1 June 2020). The completion of the questionnaire was required for participation in the study, and those who did not complete the questionnaire were excluded. The participants were asked questions about their clinical and sociodemographic characteristics, symptoms related to SARS-CoV-2 infection, comorbidities, medication use, behavioral factors, etc. All questions are shown in Supplementary Figure S1.

### 2.3. Serological Testing

Anti-SARS-CoV-2 antibodies were detected and quantified by using chemiluminescent assays for IgG (LIAISON^®^ SARS-CoV-2 S1/S2 IgG) and IgM (LIAISON^®^ SARS-CoV-2 IgM). LIAISON^®^ SARS-CoV-2 S1/S2 IgG is a quantitative test that specifically identifies antibodies against the S1 and S2 proteins of the SARS-CoV-2 spike, which are responsible for the binding and fusion of the virus to the host cell. The spike protein and its subunits are considered the main antigen targets for neutralizing antibodies. LIAISON^®^ SARS-CoV-2 IgM is a qualitative method that detects IgM antibodies against spike proteins. Both methods were performed using a LIAISON XL analyzer (DiaSorin, Saluggia, Italy). Sensitivity for IgG in patients with time elapsed since diagnosis 5–15 days is 90.7% and for patients with time elapsed since diagnosis >15 days is 97.9%. Specificity for IgG is 99%. Sensitivity for IgM in patients with time elapsed since diagnosis 5–15 days is 91.5% and for patients with time elapsed since diagnosis >15 days is 94%. Specificity for IgM is 99.2%.

### 2.4. Statistical Analysis

The results were expressed as the absolute value (n) and percentage (%) with 95% CI for categorical variables and as the mean, standard deviation (SD), median, interquartile range (IQR) (Q3-Q1) and range (minimum value, maximum value) for continuous variables. A chi-square (χ^2^) test was used to compare the associations between categorical variables, such as clinical and demographic variables, and the measured outcome was expressed as the odds ratio (OR) and its 95% CI. Continuous variables were compared with Student’s *t*-test or the Mann-Whitney test for two groups, depending on whether they had a normal or non-normal distribution. Additionally, we applied the corresponding logistic regression model for multivariate analysis of categorical variables. We considered a statistically significant difference to occur at a *p*-value < 0.05. Data analysis was performed using SPSS 27 (Statistical Package for the Social Sciences).

### 2.5. Ethics Statement

All participants enrolled in the study voluntarily, and written informed consent was required for the data to be used for analysis. Neither participation in the study nor results were reported to the participants’ employer. The study protocol was approved by the Ethical Review Board of Complejo Asistencial Universitario de Salamanca (CAUSA, Salamanca Spain CEIMc; code 2020 07 539). The procedures were carried out in accordance with the ethical standards described in the Revised Declaration of Helsinki in 2013. All clinical and epidemiological data were anonymized.

### 2.6. Role of the Funding Source

The funders facilitated data acquisition but had no role in the study design, data analysis or interpretation, or writing of the manuscript.

## 3. Results

### 3.1. Demographic Data

From the 33,178 students, professors and staff of USAL who were invited to take part in the study between 15 July 2020 and 30 October 2020, a total of 8197 (24.71%) participants were finally included (Table 1). Most of the participants were undergraduate students (5093, 62.1%), and most participants were aged 17 to 28 years (68.1%). The most represented group was technicians and administrative officers (1017 of 1210, 84.05%), followed by professors and researchers (1553 of 2300, 67.52%), undergraduate students (5093 of 20,849, 24.43%) and postgraduate students (392 of 4692, 8.35%). The mean age was 31.4 (14.5 SD) years; 66.0% of the participants were female.

### 3.2. Seroprevalence

Seropositivity for IgM and/or IgG antibodies was found in 676/8197 of the participants, corresponding to 8.25% of the sample (95% CI: 7.65–8.84), with IgM detected in 1.04% (95% CI: 0.82–1.26), IgG detected in 7.98% (95% CI: 7.39–8.57) of participants, and both in only 0.77% (95% CI: 0.58–0.96; 63/8197 participants). The highest seropositivity was found in males aged 17 to 28 years (n = 149, 9.3%, (95% CI: 7.92–10.78)) but no significant differences were found by age or sex. Seroprevalence by sex and age for each of the measured antibodies is presented in Supplementary Figure S2.

Additionally, the percentages of participants who tested positive for IgG, those who tested positive for IgM, and those who tested positive for IgG and/or IgM over time (July-October 2020) are presented in Supplementary Figure S3. Of the participants with a previous confirmed SARS-CoV-2 infection, IgG seroprevalence was 83.3% (95% CI: 66.1–100) in July, suggesting that 16.7% (95% CI: 0–33.9) of these individuals had lost the antibodies since initial infection. Additionally, 69.6% (95% CI: 62.5–76.6) of the participants with previous confirmed infection showed IgG seropositivity in October, meaning 30.4% (95% CI: 23.3–37.5) of these participants had lost the antibodies since initial infection.

### 3.3. Associations of Academic Factors with Seropositivity

We found statistically significant differences in seropositivity among academic positions (*p* = 0.020). The highest seropositivity rate occurred in the postgraduate students (9.9% (95% CI: 7.0–12.9)), followed by the undergraduate students (8.9% (95% CI: 8.1–9.7)). The lowest rate was observed in the technicians and administrative officers (6.5% (95% CI: 5.0–8.0)). The seroprevalence in the professors/researchers was 7.3% (95% CI: 6.0–8.6).

**Figure 2:**
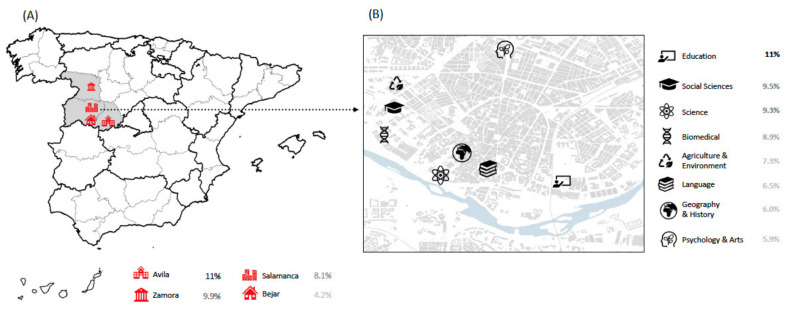
Seroprevalence of University of Salamanca: (**A**) main towns and (**B**) Salamanca city campus.

Of the cities in which the USAL campuses are located, Salamanca was home to the highest proportion of participants in the study (90.2%). The highest positivity rate was in Avila (10.8% (95% CI: 7.5–14.1)), and the lowest was in Bejar (4.2% (95% CI: 0.6–7.9)). Zamora had a positivity rate of 9.9% (95% CI: 6.8–13.0), and Salamanca had a positivity rate of 8.1% (95% CI: 7.5–8.7) (Figure 2); however, this difference among the cities was not statistically significant (*p* = 0.082). The seroprevalences of professors and students were compared in each city. Higher seropositivity rates were observed in Salamanca and Zamora, and lower rates were observed in Avila and Bejar.

**Figure 3:**
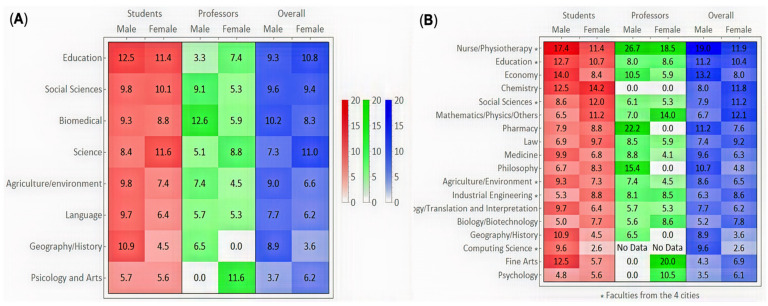
Seroprevalence in University of Salamanca Campus: (**A**) Faculties in Salamanca city, (**B**) distribution according sex and position.

Seroprevalence was analyzed for each campus in Salamanca city (Figure 2). The largest proportion of participants came from the biomedical campus (24.2%), followed by the social sciences campus (19.1%). The education, social sciences, science and biomedical campuses had seroprevalences over 8%. The highest seroprevalence was found for male students from the education campus (12.5% (95% CI: 3.8–21.2)) and male professors from the biomedical campus (12.6% (95% CI: 7.0–18.2)). Additionally, male professors from the psychology and arts campus and female professors from the geography and history campus had the lowest seroprevalence (0.0%) (Figure 3A).

There were highly significant differences in the positivity rates across the various faculties (*p* = 0.006) (Figure 3B). We observed the highest seropositivity rate in the nursing and physiotherapy faculty (13.3% (95% CI: 10.1–16.5)), followed by the education faculty (10.6% (95% CI: 8.4–12.8)). The lowest prevalence was observed in the psychology, geography and history, and languages faculties (5.7% (95% CI: 3.6–7.7), 6.1% (95% CI: 2.9–9.2) and 6.5% (95% CI: 4.5–8.5), respectively). Moreover, the highest seropositivity rate was found for male professors from the nursing and physiotherapy faculty (26.7% (95% CI: 4.3–49.1)), while the lowest percentage was found for male professors from the psychology faculty (0.0%).

### 3.4. Associations of Clinical Factors with Seropositivity

Data comparing seroprevalence by self-reported blood type and BMI are shown in Supplementary Table S1. Most of the participants had A (47.3% (95% CI: 45.9–48.6)) or O (40.3% (95% CI: 39.0–41.7)) blood type, and no significant differences in seropositivity were found among blood types. BMI ranged from 18.5 to 24.9 (67.0% (95% CI: 66.0–68.1)); logistic regression analysis revealed no significant relationship between BMI and seropositivity.

**Figure 4:**
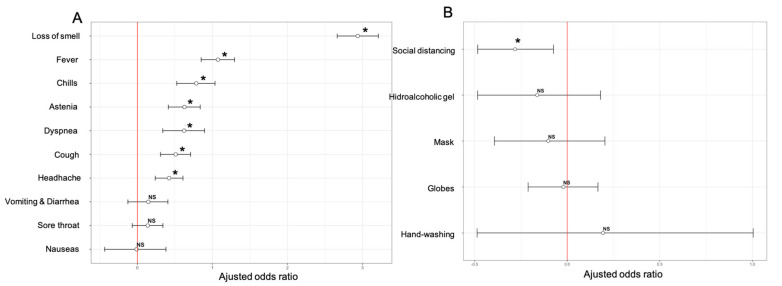
Adjusted odds-ratio and 95% confidence intervals for community exposure associated with main symptoms (**A**) and hygiene measures (**B**) with COVID-19. * *p* < 0.05. NS: Non-significant.

Seroprevalence was also studied according to the self-reported presence of symptoms, diseases and drug prescriptions. Among the 676 seropositive participants, 153 (22.6%) were asymptomatic and 523 (77.4%) were symptomatic. Figure 4A shows ORs for the associations of seropositivity with the main symptoms. Loss of smell (5.3% vs. 54%; OR 20.69 (15.95–26.89)) and fever (5.9% vs. 16%; OR 3.02 (2.45–3.72)) were the symptoms most strongly associated with seropositivity. No significant associations were found between seropositivity rates and the overall frequencies, comorbidities or prescriptions of corticosteroids, inhalers or antihistamines.

### 3.5. Associations of Lifestyle and Behavioral Factors with Seropositivity

The participants’ smoking status and alcohol consumption were also studied. No significant difference in seropositivity was found between alcohol consumers and non-consumers (8.1% for alcohol consumers vs. 8.3% for non-consumers; OR 0.93 (95% CI: 0.67–1.29)). However, a difference was found between smokers and non-smokers, and interestingly, seropositivity more strongly associated with non-smoking (5.2% for current smokers vs. 8.6% for non-smokers; OR 0.57 (95% CI: 0.42–0.79)).

Seroprevalence according to hygiene measures is presented in Figure 4B. We identified a significantly decreased seroprevalence in people who adopted social distancing (*p* = 0.0007), but no other major differences. Table 2 shows the association of the type of residence and the numbers of cohabitants and pets with seroprevalence. There were significant differences in seroprevalence between participants with and without household exposure to SARS-CoV-2 (*p* = 0.0000). In the student group, there were no differences in seroprevalence between those who lived in private homes and those who lived in dormitories.

## 4. Discussion

Our cross-sectional study was carried out between June and October 2020 using chemiluminescent assays for antibody detection and a questionnaire. The overall objective of our study was to carry out a comprehensive study of the university community to guide strategies to mitigate possible cases of COVID-19 at USAL for safe reopening in the 2020/2021 academic year. We analyzed the demographic, academic, clinical and lifestyle and behavioral factors associated with COVID-19 in a university sample, with a high participation rate. It was found that (i) the overall seroprevalence of anti-SARS-CoV-2 antibodies (IgG and/or IgM) was 8.25%—this rate is similar to that in studies of larger communities [14]; (ii) antibody seropositivity decreased over time; (iii) there was a higher seroprevalence in students and professors in health-related campuses and faculties than in other campuses and faculties; (iv) the seroprevalence was similar across campuses, but there were highly significant differences among faculties; (v) asymptomatic status was observed in 22.6% of the seropositive participants and loss of smell was the main symptom associated with antibody detection; and vi) the only hygiene measure associated with lower seroprevalence was social distancing.

Seroprevalence studies are currently being implemented worldwide, as they are considered a valuable tool to reveal the extent of SARS-CoV-2 infection via the estimation of the proportion of the population exposed to the virus. To the best of our knowledge, this is the largest study describing the prevalence of SARS-CoV-2 in an academic population in Europe. The seroprevalence of the university community observed in our study and that previously found for the general population were very similar [14]. However, the USAL seroprevalence rate was higher than the seroprevalence rates of other academic communities, such as the University of Southern California [10] and the University of Pennsylvania [11] in the USA, the University of Athens in Greece [12] and the University of Alicante in Spain (8) (2.6–5.5%) but lower than that of the University of Sergipe in Brazil (22.5%) [9]. These differences could be due to the following: (i) some studies included a smaller number of participants; (ii) the previous studies were carried out exclusively with students; (iii) the serological tests used varied across studies with different levels of sensitivity; and (iv) studies were performed over different periods of time.

One main finding of this study was a difference in seropositivity between students and university staff. The highest rate was found in postgraduate students followed by undergraduate students, and the lowest rate was observed in technicians and administrative officers. Students’ large social networks could be a primary cause of these results. We initially expected that students could be responsible for the spread of infection in our region. However, our results indicate that student communities had exhibited more protective behavior against the spread of the pandemic than other groups.

In addition, while we found that seropositivity did not significantly differ across cities, the highest seroprevalence was found in the participants from Avila, probably due to the proximity of this town to the capital of Spain, Madrid. It is well known that Madrid had a higher seroprevalence in the first wave of the pandemic than other Spanish cities due to the centrifugal spread of the virus [3]. Social factors, such as the population structure and poverty, which were not considered in our study, might also explain the higher prevalence in Avila.

In terms of subject area, our comparison of the seroprevalence across Salamanca campuses showed the highest seropositivity rates for students from the education campus and male professors from the biomedical campus. The first clinical cases detected at USAL were in the education faculty, and the PCR technique was not systematically used to identify students with contact with the index patient, which allowed the infection to spread. In addition, higher seroprevalence was found in professors from biomedical campuses, particularly in the nursing and physiotherapy faculty due to their interactions with hospital environments.

We also investigated the association of clinical and lifestyle factors, such as comorbidities, BMI, blood type, smoking and alcohol consumption, with seropositivity. Previous research has shown that comorbidities occur with SARS-CoV-2 infection in approximately half of inpatients. Hypertension was found to be the most common comorbidity, followed by obesity, diabetes and coronary heart disease [15]. Moreover, in a different study, obesity and adiposity-related diseases were shown to be clearly related to worse disease evolution [16]. In our study, no differences in seroprevalence according to weight (represented as BMI) and groups of diseases consistently linked to the prescription of certain medications were observed. Patients with a smoking history had a higher likelihood of developing more severe symptoms of COVID-19 disease than non-smokers. However, data on whether COVID-19 has a greater incidence in smokers than non-smokers have thus far been contradictory and inconclusive [17]. Surprisingly, our data showed tobacco use to be a protective factor, demonstrating the need for more studies to clarify the role of smoking in the incidence of COVID-19. Interestingly, previous research showed that the ABO blood group was associated with SARS-CoV-2 infection and survival [18]. Group A has been found to be more common, while group O has been found to be less common among infected individuals. Moreover, blood group O has shown lower mortality than the other ABO blood groups. In our study, the ABO blood group did not show any relationship to seroprevalence.

Furthermore, we examined the association of various symptoms with seropositivity. The most common symptoms among young SARS-CoV-2 patients were previously found to involve the ear and nose [19]. In our cohort, which was composed primarily of students, the main clinical manifestation linked with higher seroprevalence was loss of smell. Interestingly, 22.6% of the participants who presented antibody positivity did not report any symptoms. This finding suggests that asymptomatic infection is relatively common in a healthy population. Thus, among asymptomatic individuals, infections could resolve spontaneously without complications, as occurs in other coronavirus infections. Therefore, the rapid identification of asymptomatic individuals is essential to control the spread of infection. Moreover, clinical characteristics could influence the real prevalence of this disease. Additionally, aspects of infection, such as immunity, reinfection and cross-reactivity with human endemic coronavirus, are not yet known [20].

Governments across the world have implemented a wide range of measures to mitigate the spread of SARS-CoV-2 infection, but the optimal non-pharmaceutical strategies are not entirely clear [21]. Our findings highlight differences between adults in the academic community who received positive SARS-CoV-2 test results and those who received negative SARS-CoV-2 test results. Our data showed that among various hygiene measures, such as the use of hydroalcoholic gel, masks and gloves, only social distancing was associated with a significantly decreased seroprevalence. Continued assessment of the activities and exposure of communities, schools and workplaces during reopening is important. Exposure and activities where mask use and social distancing are difficult to maintain, including going to locations that offer on-site eating and drinking, might be important risk factors for SARS-CoV-2 infection. Hence, implementing safe practices to reduce exposure to SARS-CoV-2 during on-site eating and drinking should be considered to protect customers, employees and communities and to slow the spread of COVID-19 [22].

Regarding the place of transmission, our data showed significant differences between participants with and without household exposure to SARS-CoV-2. These results are consistent with other reports suggesting that households are the principal place of transmission [23]. Interestingly, we also noted that in the student group, there were no differences between those who lived in private homes and those who lived in dormitories, which is in contrast to the assumption that colleges would be environments with a higher risk of infection since they are spaces characterized by a greater amount of social interaction.

Seroprevalence over time is the main indicator of the maintenance of specific antibodies against SARS-CoV-2. Our results were similar to those of other studies that showed a decrease in IgG antibodies over time [24]. The epidemiological impact of the decrease in seroprevalence over time in academic communities must be elucidated.

Several limitations of this study must be considered. By design, this study was carried out in a specific population. Thus, the results cannot be extrapolated directly to the general population. The serological tests we used in this study could also be a limitation. However, chemiluminescent assays were shown to have higher sensitivity and specificity rates than other methods [25]. Additionally, data were obtained through a self-report questionnaire completed by the participants. Neither ethnicity nor income data were collected, preventing the analysis of previously demonstrated associations with COVID-19 positivity [26].

In summary, our analysis of more than 8100 USAL community members estimated the exposure of members of this community to SARS-CoV-2, revealing approximately 8% seroprevalence from July–October 2020 and a higher prevalence in students than in university staff. Our findings suggest that there is no need for tailored measures for USAL members who should comply with public health measures, especially the maintenance of social distancing, as well as implement new measures, such as vaccination.

## Figures and Tables

**Table 1:** Main data according to the different demographic variables selected: age, sex, categories, location campus and faculty.

Variables		*n* = 8197 (%)
**Age**		
Mean ± SD; years		31.4 ± 14.5
Population	17–28	5503 (68.1%)
	29–39	486 (6.0%)
	40–49	606 (7.5%)
	50–59	1039 (12.9%)
	60–76	441 (5.5%)
**Sex**		
Male		2709 (34.0%)
Female		5248 (66.0%)
**Position**		
Students	Undergraduate	5093 (62.1%)
	Postgraduate (Master and PhD)	392 (4.8%)
Professors		1553 (18.9%)
Technicians and Administrative Officers		1017 (12.4%)
Others		142 (1.7%)
**Salamanca University Campus Map**		
Salamanca		7390 (90.2%)
Zamora		355 (4.3%)
Avila		334 (4.1%)
Bejar		118 (1.4%)
**Salamanca University Campus**		
Agriculture and Environment		160 (2.2%)
Biomedical		1791 (24.2%)
Education		456 (6.2%)
Geography and History		215 (2.9%)
Language		601 (8.1%)
Psychology and Arts		580 (7.8%)
Science		687 (9.3%)
Social Sciences		1408 (19.1%)
Others		1492 (20.2%)

**Table 2:** Seroprevalence relationship with dwelling and exposure.

Dwelling and Exposure		*n* = 7034 (%)	Seropositivity	
			*n* (%)	*p*-Value
**Residence**	Professors	1549 (22.0%)	114 (7.4%)	0.133
	Student private house	5039 (71.6%)	449 (8.9%)	
	Student colleges	446 (6.3%)	42 (9.4%)	
**Life with animals**	Yes	1867 (22.8%)	138 (7.4%)	0.127
	No	6330 (77.2%)	538 (8.5%)	
**Exposure**	Household	637 (8.0%)	189 (29.7%)	0.000
	No household	7049 (88.2%)	436 (6.2%)	
	Not know	302 (3.8%)	31 (10.3%)	
**Household Exposure**	Colleges	38 (6.0%)	13 (34.2%)	0.583
	Private house	599 (94.0%)	176 (29.4%)	

## Supplementary Materials

The following are available online at https://www.mdpi.com/article/10.3390/jcm10153214/s1, Figure S1: Questionnaire, Figure S2: Seroprevalence by sex and age group, Figure S3: Seroprevalence according to the time of the serology, Table S1: Seroprevalence of IgG & IgM antibodies according to blood types and Body Mass Index (BMI).

## Appendix A. DIANCUSAL Team (Alphabetical Order)

1.	Judy	Aala
2.	Elisa	Acosta de la Vega
3.	Álvaro	Aguado Muñiz
4.	Alberto	Alén Andrés
5.	Raquel	Álvarez Lozano
6.	Luis Manuel	Álvarez Oricheta
7.	Roberto	Arévalo Pérez
8.	Elisabeth	Arias Gómez
9.	Samuel	Barbero Garrote
10.	Beatriz María	Bermejo Gil
11.	Enrique	Blanco Peláez
12.	Noelia	Bullón
13.	Esther	Caballero Salvador
14.	Laura	Cabrero
15.	Juan Carlos	Calderón
16.	Rubén	Cañizares Sanchez
17.	Cristina	Carbonell Muñoz
18.	Elena	Carnicero Antón
19.	Raquel	Carnicero Izquierdo
20.	Ana	Carranza de Frutos
21.	Carlos	Carrera Tomás
22.	Laura	Cid Mendes
23.	Sonia	Clavero Sánchez
24.	Clara Isabel	Colino Gandarilla
25.	Victoria	Coral Orbes
26.	Diego	Cotobal García
27.	Beatriz	Crego Vicente
28.	Luis	de Alfonso Vazquez
29.	Manuel	De la Cruz Garcinuño
30.	María Teresa	De la Puente Sanz
31.	Francisco Javier	Derteano Ortiz de Artiñano
32.	David	Eguiluz López
33.	Daniel	Encinas Sanchez
34.	Carlos	Estévez Colmenero
35.	Begoña	Febrer Sendra
36.	Helena	Fernández Cabrera
37.	Adolfo	Fernández Sánchez
38.	Pedro	Fernández Soto
39.	Javier	Flores Fraile
40.	Manuel	Fuentes García
41.	Andrea	Fuentes Gordillo
42.	Raúl	Fuentes Martín
43.	Ana Isabel	Galán Hernández
44.	Juan	García-Bernalt Diego
45.	Lucía	García Aparicio
46.	Carlos	García Cabezas
47.	Vega	Garcia Cirilo
48.	Raquel	Garcia López
49.	María de los Ángeles	García Pascua
50.	José Ángel	García Pedraza
51.	Paula	García Vallés
52.	Nerea	Gestoso Uzal
53.	Mariona	Gil Llagostera
54.	Sonia	Gómez Gaspar
55.	María Isabel	González Flores
56.	Susana	González Manzano
57.	José	Gordo Gonzalo
58.	Oscar	Gorgojo Galindo
59.	Carlos	Gutiérrez Cerrajero
60.	Rosa	Hermosa Prieto
61.	Reyes	Hernández
62.	Isabel María	Hernández de la Fuente
63.	Luis Manuel	Hernández Medina
64.	Nieves	Hernández San Antonio
65.	Santiago	Herrero González
66.	Luis	Jiménez Jurado
67.	Rosa	Juana Tejera Pérez
68.	Paula	Linde Leiva
69.	Inés	Llamas Ramos
70.	Julio	López Abán
71.	Amparo	López Bernus
72.	Joaquín F	López Marcos
73.	Noelia	López Velázquez
74.	Antonio	López-Valverde Centeno
75.	Nansi	López-Valverde Hernández
76.	María	Lorenzo Santiago
77.	Paloma	Malmierca Román
78.	Elvira	Manjón Pérez
79.	Sergio	Manso Hierro
80.	Laura	Márquez Arcos
81.	Abel Jesús	Martel Martel
82.	Juan Carlos	Martín Corral
83.	Alba	Martín del Rey
84.	Raquel	Martín Fernández
85.	Elena	Martín González
86.	Alba	Martín Hernández
87.	Daniel	Martín Hidalgo
88.	Manuel	Martín Morales
89.	Ana María	Martín Nogueras
90.	Ana	Martín Suarez
91.	Andrea	Martín Tomé
92.	María	Martínez Ferradal
93.	Alba	Mata Caballero
94.	Diego	Matellán Alonso
95.	Laura	Mateos Sánchez
96.	Francisco José	Matos
97.	Marta	Mayo Caballero
98.	Isabel	Méndez Hernández
99.	Roberto	Méndez Sánchez
100.	Alba María	Merino Expósito
101.	Adrián	Miguélez Martínez
102.	Elly	Mondolis
103.	Cristina	Mora González
104.	Carlos	Moreno Dorado
105.	Raquel	Moreno García
106.	Mirian	Moreno Ramos
107.	Jorge	Moreno Teniente
108.	Daniel	Muñoz Reyes
109.	Elena	Naranjo Bueno
110.	Verónica	Navarro Santamaría
111.	Cecilia	Oliva Mangas
112.	Paula	Oramas Padrón
113.	Olga	Ortuño López
114.	María	Ovejero Sánchez
115.	María	Oviedo Madrid
116.	Josué	Pendones Ulerio
117.	Sara	Peral Garrido
118.	Ana	Perera Gregorio
119.	Leyre	Pérez Hernández
120.	Laura	Pérez Huerga
121.	Sade	Pérez López
122.	Daniel	Pérez Martin
123.	Daniela	Pérez Ramos
124.	Fátima	Pérez Robledo
125.	Ángel	Pindado Pérez
126.	Roció	Pindado Saez
127.	Carlos Rafael	Pires Baltazar
128.	Olga	Pozas Flores
129.	Isabel	Redero Sanchón
130.	María José	Rodrigo Gonzalo
131.	Clara	Rodrigo Pérez
132.	Beatriz	Rodríguez Alonso
133.	Carlos	Rodríguez Carneiro
134.	Celia	Rodríguez Tudero
135.	Melanie	Ruiz Navarro
136.	Enrique	Sánchez Carrasco
137.	Myriam	Sánchez Díaz
138.	Ana M	Sánchez Fernández
139.	Daniel	Sánchez González
140.	Javier	Sánchez Montejo
141.	Carmen	Sánchez Sánchez
142.	María	Sánchez Tabernero
143.	Alicia	Sanjosé Crespo
144.	Guillermo	Santabrígida Oreja
145.	Laura	Santos Gómez
146.	Elena	Santos Hernandez
147.	Carmen	Santos Marcos
148.	María	Santos Plaza
149.	Cristina	Sanz Cuesta
150.	Rosa	Sepúlveda Correa
151.	Teresa	Sereno Mateos
152.	Susana	Sudon Pollo
153.	Vladut Alexandru	Tanase Iosub
154.	Javier	Tascón Romero
155.	José	Tortosa Cámara
156.	Elena	Varas Martín
157.	Ana	Vicente García
158.	Lidia	Vicente Medina
159.	Laura	Vicente Vicente
160.	Carmen	Vieira Lista
161.	Paula	Vigario Calaco
162.	Elena	Villanueva Sánchez
163.	Cristina	Villaoslada Fuentes
164.	Aranzazu	Zarzuelo Castañeda
165.	Pilar	González Arrieta
166.	Rosa Isabel	Sánchez Alonso
167.	Mª del Pino	Mendez Arroyo
168.	David	Martín Fernandez
169.	Laura	del Rio Sanz
170.	Pilar	González Barez
171.	Jesús	Martín González
172.	Jorge	García Pindado
173.	Vega	Angulo Sánchez
