# Supplementary material for: Seroprevalence of SARS-CoV-2 Antibodies and Factors Associated with Seropositivity at the University of Salamanca: The DIANCUSAL Study
Source: J Clin Med. 2021 Jul 21;10(15):3214. doi: 10.3390/jcm10153214 (PMC8348112; doi:10.3390/jcm10153214)
Supplement: Supplementary file 1 [file jcm-10-03214-s001.zip › jcm-1244683-suppl/Supplementary_1_figure.pdf]

Supplementary Figure 1

QUESTIONNAIRE

Welcome to the DIANCUSAL clinical-epidemiological questionnaire

Weight and height data

Height (in cm.) *

Weight (in kg.)*

1. Information about your home during the 20/21 academic year

Type of residence* Apartment / University Residence

If you live in an apartment, square meters of your home

If you live in a residence, Name of the university residence

Place of your habitual residence* **Postal Code**

Place of confinement* **Cod. Confinement postcard**

Number of habitual partners*

Number of partners during confinement*

Pets **Number**
YES / NO Species

2. Clinical data

Toxic Habits No / Smoker / Alcohol / Other drugs

Medical antecedents

Blood group A / B / AB / O / No I know

Diseases [write another disease] / Arterial Hypertension (HBP) / Diabetes Mellitus (DM) / Cardiovascular Risk Asthma / Chronic Obstructive Pulmonary Disease / Immunosuppression / Cardiovascular Pathology / Chronic kidney failure

Number of chronic medications

Types of medications

**What protective measures have you used**	None / Social withdrawal / Gloves / Gel / Handwashing Mask

3. COVID Diagnosis Have

**you been diagnosed with COVID?**	YES / NO
**If the previous answer is affirmative, have you required hospitalization?**	YES / NO
**Date of admission**	
**Place of hospitalization**	
**Date of discharge**	
**Briefly explain the reasons for admission**	
**Clinical Evolution**	

4. If you have not been diagnosed with COVID, have you had any of these symptoms?

**Do you think you may have had COVID?**	YES / NO / Don't know / can
**Fever**	YES / NO / Don't know / can
**Cough**	YES / NO / Don't know / can't know / can
**Sore throatSevere**	YES / NO / Don't know / can
**Chills**	YES / NO / Do
**Tiredness**	YES / NO / Don't know / can
**Feeling short of breath**	YES / NO / Don't know / can
**Headache**	YES / NO / Don't know / can
**Nausea**	YES / NO / Don't know / can
**Vomiting and / or diarrhea**	YES / NO / No know / may
**Sudden loss of sense of smell or taste**	YES / NO / Don't know / may

5. COVID-19 diagnosis of a COVID-19:any

**Hascohabitant been diagnosed with COVID?**	YES / NO / Don't know / can
**how many?**	
**Age / Ages**	
**Degree of familiarity**	
**Have they required hospitalization?**	YES / No / Don't know

6. Even though no COVID-19 has been diagnosed with COVID, do you know if they have had any of these symptoms?

**Do you think that a partner may have had COVID even though it has not been confirmed?**	YES / NO / Don't know / can
**Fever**	YES / NO / Don't know / can
**Cough**	YES / NO / Don't know / cann't know / can
**Sore throatSevere**	YES / NO / Don't know / can
**Chills**	YES / NO / Do
**Tiredness**	YES / NO / Don't know / can
**Feeling short of breath**	YES / NO / Don't know / can
**Headache**	YES / NO / Don't know / can
**Nausea**	YES / NO / Don't know / can
**Vomiting and / or diarrhea**	YES / NO / No know / can
**sudden loss of sense of taste or smell**	YES / NO / Do not know / can
