# Supplementary material for: Seroprevalence of SARS-CoV-2 Antibodies and Factors Associated with Seropositivity at the University of Salamanca: The DIANCUSAL Study
Source: J Clin Med. 2021 Jul 21;10(15):3214. doi: 10.3390/jcm10153214 (PMC8348112; doi:10.3390/jcm10153214)
Supplement: Supplementary file 1 [file jcm-10-03214-s001.zip › jcm-1244683-suppl/Supplementary_1_table.pdf]

Supplementary table 1. Seroprevalence of IgG & IgM antibodies according to blood types and Body Mass Index (BMI)

Blood type & Body mass index	N=8197 (%)	IgG+			IgM+			IgG+ and/or IgM+		
		N=n (%)	p-value		N=n (%)	p-value		N=n (%)	p-value	
Blood type	N=5210 (%)									
O	2102 (40.3%)	159 (7.6%)	0.147		23 (1.1%)	0.752		167 (7.9%)	0.110	
A	2463 (47.3%)	221 (9.0%)			33 (1.3%)			227 (9.2%)		
B	454 (8.7%)	30 (6.6%)			4 (0.9%)			30 (6.6%)		
AB	191 (3.7%)	19 (9.9%)			3 (1.6%)			21 (11.0%)		
Body Mass Index (BMI)	N=8183 (%)	N=n (%)	OR 95% CI	p-value	N=n (%)	OR 95% CI	p-value	N=n (%)	OR 95% CI	p-value
<18.5	560 (6.8%)	41 (7.3%)	1.009 [0.988,1.029]	0.415	4 (0.7%)	1.013 [0.960,1.069]	0.637	43 (7.7%)	1.006 [0.986,1.027]	0.541
18.5-24.9	5486 (67.0%)	443 (8.1%)			54 (1.0%)			459 (8.4%)		
25-29.9	1665 (20.3%)	133 (8.0%)			25 (1.5%)			137 (8.2%)		
30-34.9	385 (4.7%)	31 (8.1%)			2 (0.5%)			31 (8.1%)		
>35	87 (1.1%)	6 (6.9%)			0 (0.0%)			6 (6.9%)		
