# Supplementary material for: Seroprevalence of SARS-CoV-2 Antibodies and Factors Associated with Seropositivity at the University of Salamanca: The DIANCUSAL Study
Source: J Clin Med. 2021 Jul 21;10(15):3214. doi: 10.3390/jcm10153214 (PMC8348112; doi:10.3390/jcm10153214)
Supplement: Supplementary file 1 [file jcm-10-03214-s001.zip › jcm-1244683-suppl/Supplementary_2_figure.pdf]

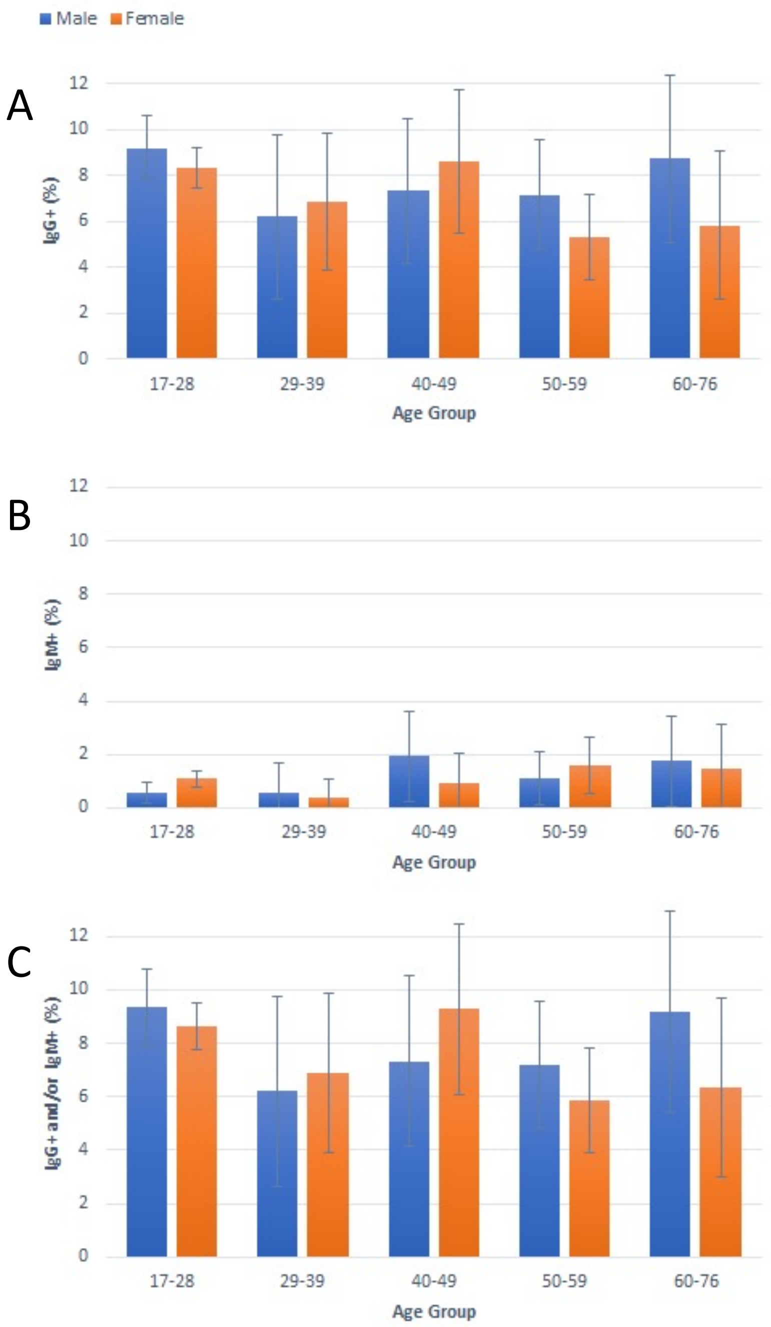

Supplementary figure 2. Seroprevalence by sex and age group: A) IgG antibodies, B) IgM antibodies and C) IgG and/or IgM antibodies
