# Supplementary material for: Seroprevalence of SARS-CoV-2 Antibodies and Factors Associated with Seropositivity at the University of Salamanca: The DIANCUSAL Study
Source: J Clin Med. 2021 Jul 21;10(15):3214. doi: 10.3390/jcm10153214 (PMC8348112; doi:10.3390/jcm10153214)
Supplement: Supplementary file 1 [file jcm-10-03214-s001.zip › jcm-1244683-suppl/Supplementary_3_figure.pdf]

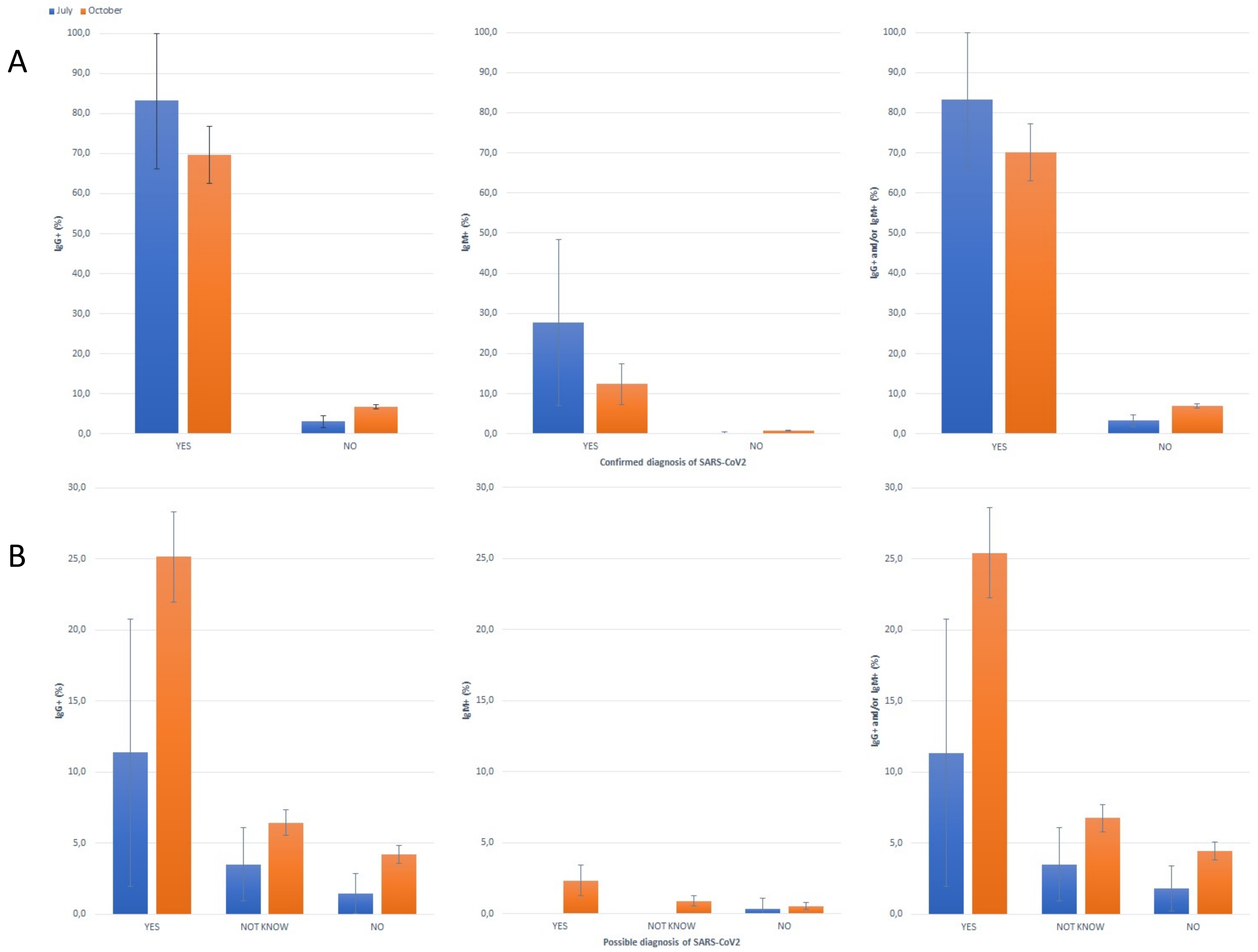

Supplementary figure 3. Seroprevalence according to the time of the serology A) Confirmed diagnosis of SARS-CoV2, B) Possible diagnosis of SARS-CoV2
